# Supplementary material for: Interactions between TTYH2 and APOE facilitate endosomal lipid transfer
Source: Nature. 2025 Jun 25;644(8075):273–9. doi: 10.1038/s41586-025-09200-x (PMC12328215; doi:10.1038/s41586-025-09200-x)
Supplement: Supplementary file 1 — Supplementary Figs. 1–9. [file 41586_2025_9200_MOESM1_ESM.pdf]

nature portfolio

https://doi.org/10.1038/s41586-025-09200-x

Supplementary information

Interactions between TTYH2 and APOE facilitate endosomal lipid transfer

In the format provided by the authors and unedited

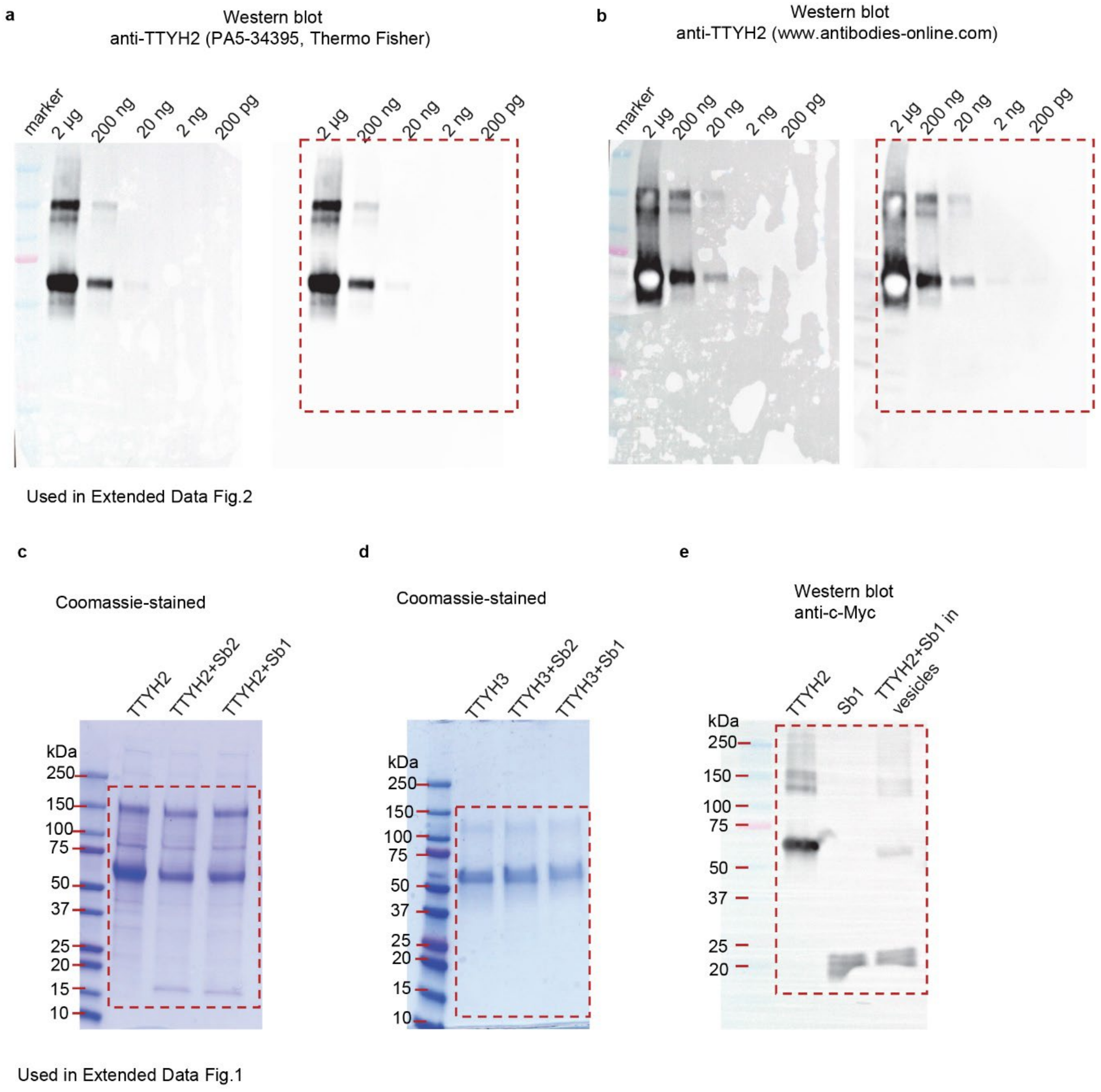

Supplementary Fig. 1. | Uncropped SDS-PAGE gels and Western blots from Extended Data Figs. 1 and 2. Western blots for Extended Data Fig. 2f showing recognition of purified TTYH2 for anti-TTYH2 PA5-34395 antibody obtained from Thermo Fisher (**a**) and for anti-TTYH2 antibody obtained from www.antibodies-online.com (**b**). Coomassie-stained SDS-PAGE gels for Extended Data Fig. 1a,b showing peak fractions from the binding tests of Sb1 and Sb2 to TTYH2 (**c**) and TTYH3 (**d**). A Western blot for Extended Data Fig.1i showing the presence of the TTYH2 band in the cell-derived vesicles isolated using Sb1.

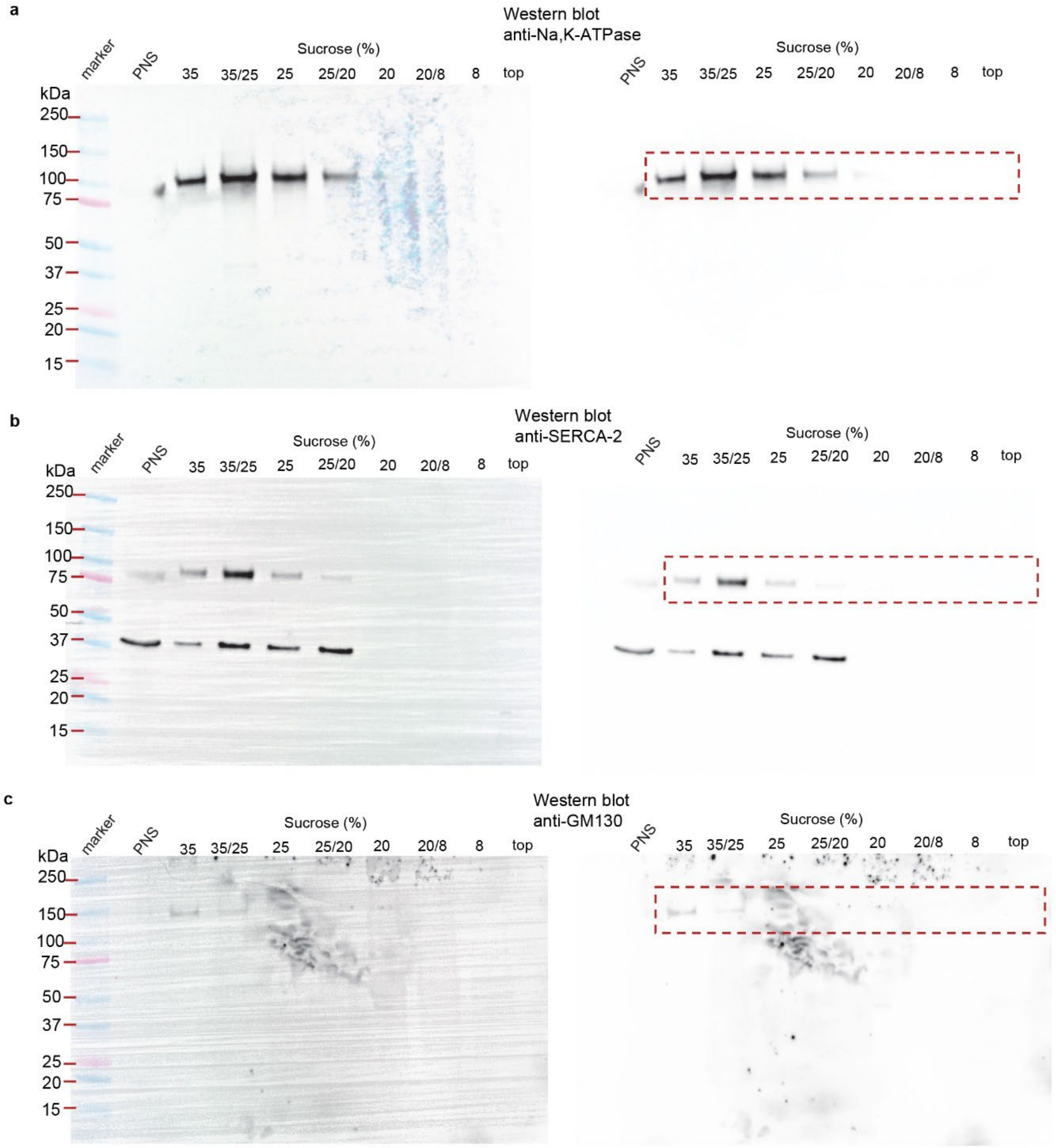

Used in Extended Data Fig.2. Fractionation HEK293 cells.

Supplementary Fig. 2. Uncropped Western blots from Extended Data Fig. 2. Western blots for Extended Data Fig. 2d obtained using the following antibodies (Thermo Fisher): **a**, rabbit anti-Na,K-ATPase (ST0533), **b**, rabbit anti-SERCA (JM10-20), **c**, rabbit anti-GM130 (ARC0589).

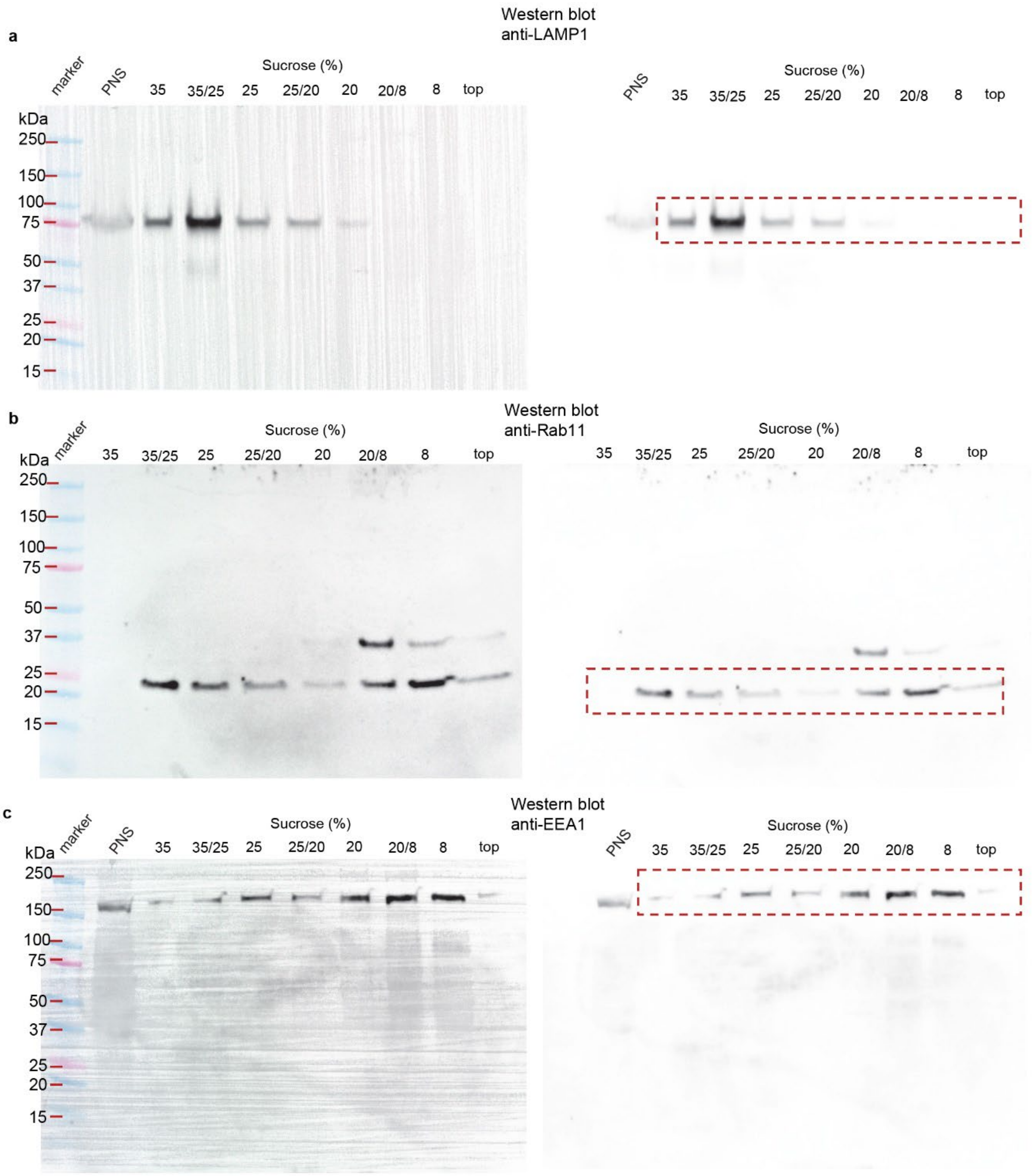

Used in Extended Data Fig.2. Fractionation HEK293 cells.

Supplementary Fig. 3. Uncropped Western blots from Extended Data Fig. 2. Western blots for Extended Data Fig. 2d obtained using the following antibodies (Thermo Fisher, unless indicated): **a**, rabbit anti-LAMP1 (107), **b**, rabbit anti-Rab11 (20229-1-AP; Proteintech), **c**, rabbit anti-EEA1 (F.43.1).

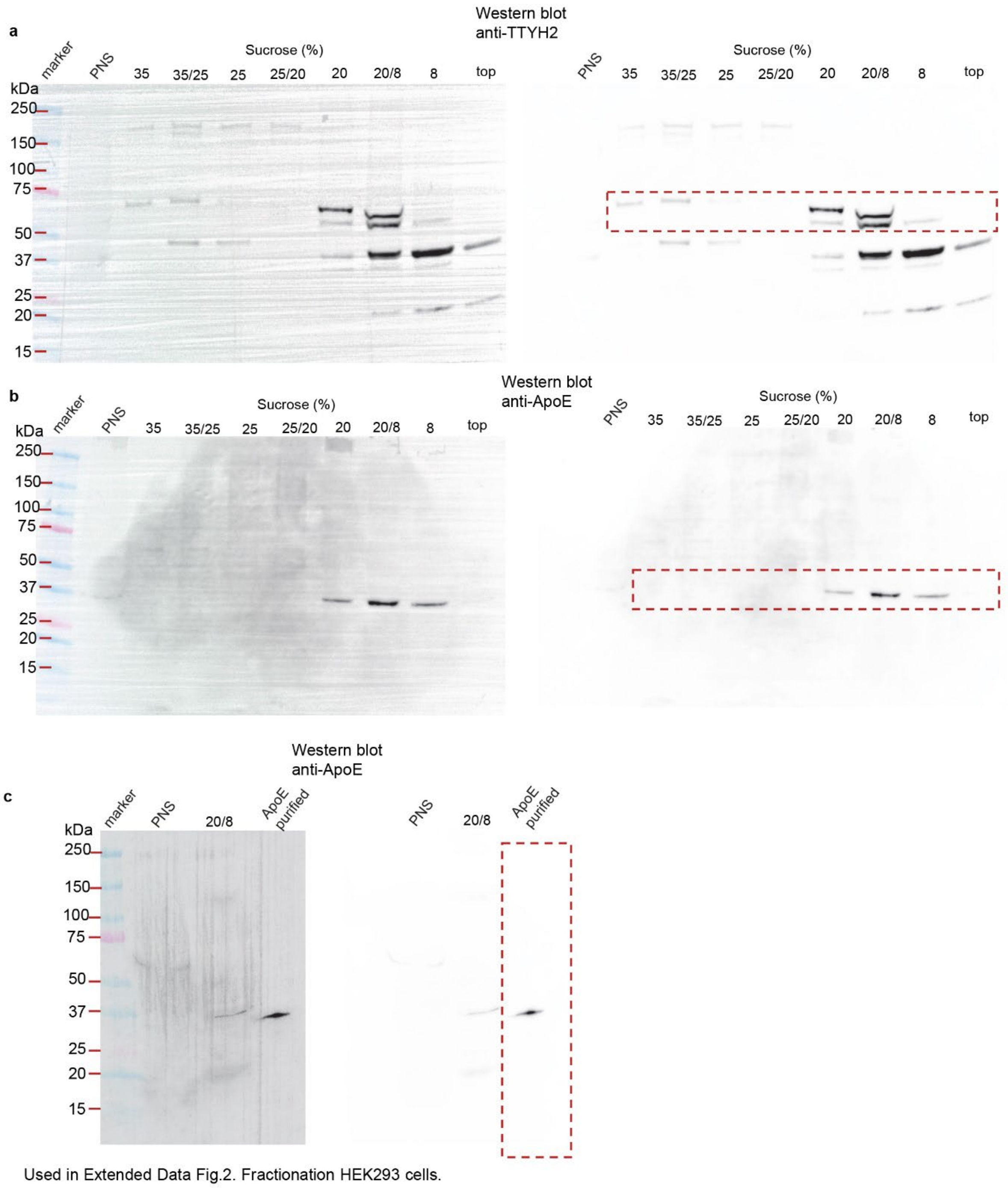

Supplementary Fig. 4. Uncropped Western blots from Extended Data Fig. 2. Western blots for Extended Data Fig. 2d obtained using the following antibodies (Thermo Fisher): **a**, rabbit anti-TTYH2 (PA5-34395), **b**, rabbit anti-ApoE (16H22L18), **c**, recognition of purified ApoE for anti-ApoE antibody (16H22L18).

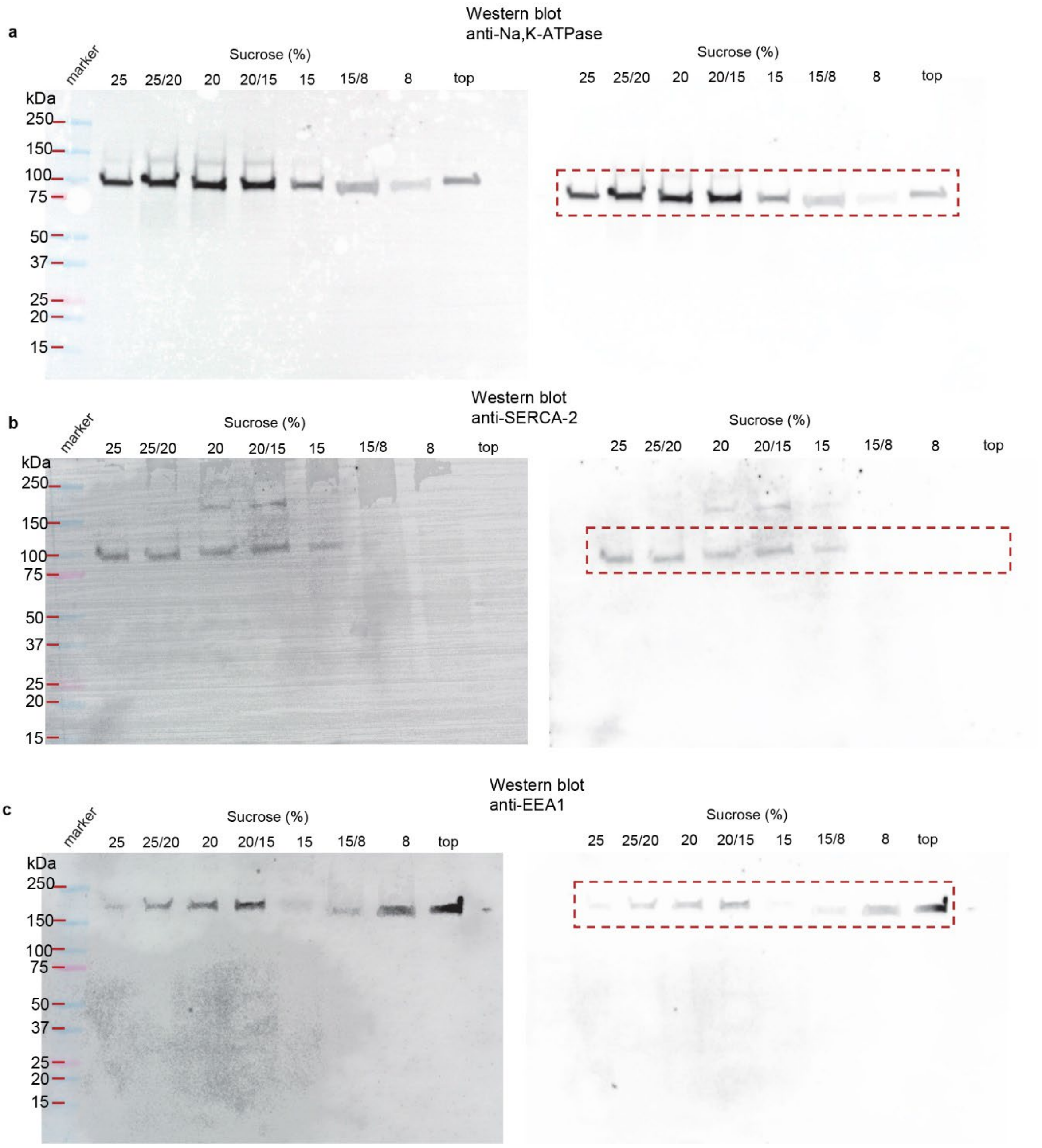

Used in Extended Data Fig.2. Fractionation N2A cells.

Supplementary Fig. 5. Uncropped Western blots from Extended Data Fig. 2. Western blots for Extended Data Fig. 2e obtained using the following antibodies (Thermo Fisher): **a**, rabbit anti-Na,K-ATPase (ST0533), **b**, rabbit anti-SERCA (JM10-20), **c**, rabbit anti-EEA1 (F.43.1).

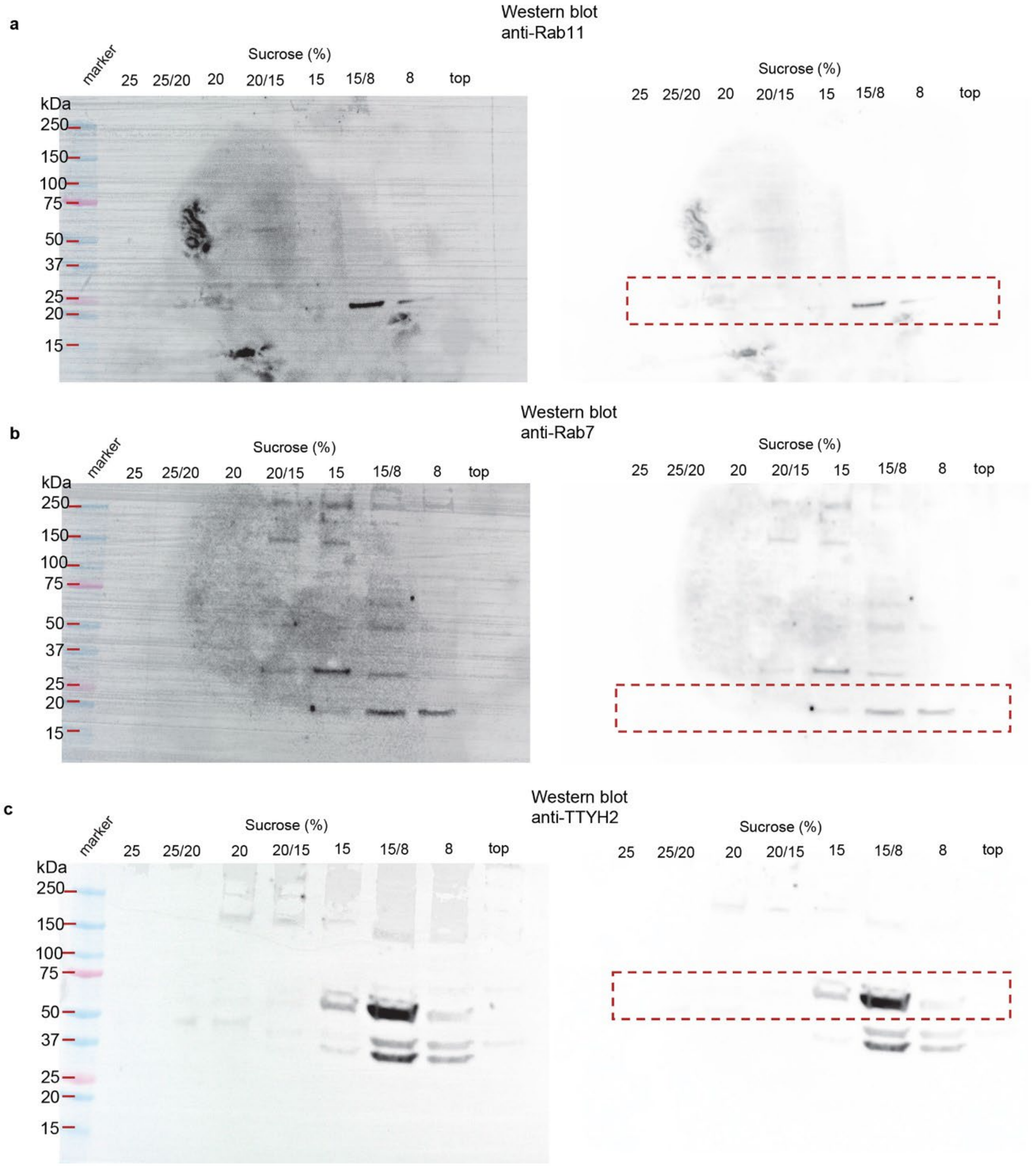

Used in Extended Data Fig.2. Fractionation N2A cells.

Supplementary Fig. 6. Uncropped Western blots from Extended Data Fig. 2. Western blots for Extended Data Fig. 2e obtained using the following antibodies (Thermo Fisher, unless indicated): **a**, rabbit anti-Rab11 (20229-1-AP; Proteintech), **b**, rabbit anti-Rab7 (PA5-23138), **c**, rabbit anti-TTYH2 (PA5-34395).

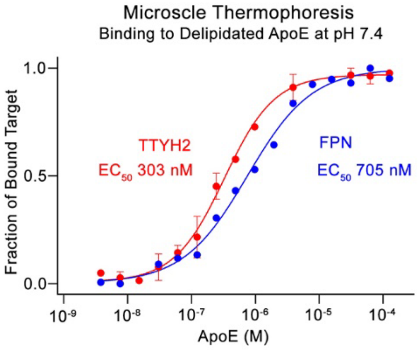

Supplementary Fig. 7. | Microscale Thermophoresis. Microscale Thermophoresis (MST) was used to study the interaction between TTYH2 and ApoE. TTYH2 and the metal ion transporter ferroportin (FPN) were purified in the detergent GDN. Both proteins were fluorescently labeled using the MO-LO11 protein labeling kit RED-NHS 2nd Generation (NanoTemper) according to manufacturer's instructions. ApoE was titrated in a 16-step 1:2 dilution series using buffer containing 10 mM HEPES pH 7.4, 200 mM NaCl and 50 µM GDN, while the membrane protein concentration was kept at 10 nM. The protein mixtures were incubated on ice for 5 min before loading it into the standard Monolith NT.115 capillaries (NanoTemper). MST signals were measured using a Monolith NT.115 instrument (NanoTemper) at 25 ºC using 20% LED power and 80% MST power. ApoE binding to TTYH2 was measured twice, and its binding to FPN once as a negative control. EC_{50} values were obtained from a fit of a single site binding isotherm to the normalized MST temperature jump fluorescence values in GraphPad Prism 10. The titration of delipidated ApoE to TTYH2 shows a saturable binding event with an EC_{50} of 303 nM. A similar binding with somewhat lower affinity was also observed for the Fe^{2+}-transporter Ferroportin (FPN), presumably reflecting non-specific interactions.

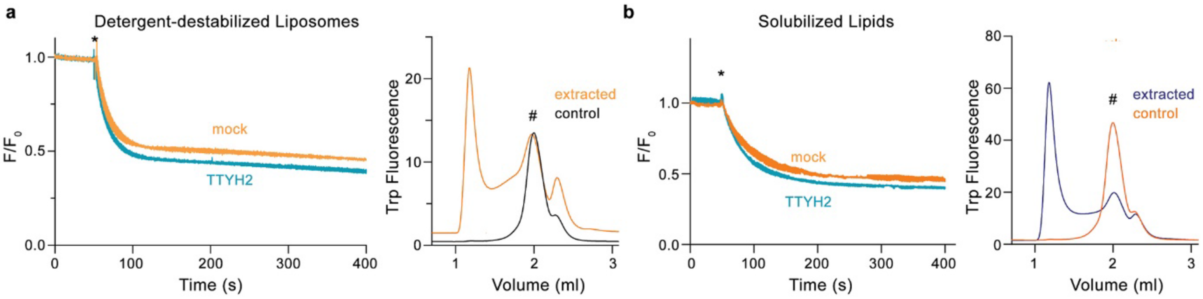

Supplementary Fig. 8. | Lipid Scrambling Assay. Assay of lipid scrambling in proteoliposomes containing TTYH2 reconstituted from, **a**, preformed and destabilized liposomes and, **b**, solubilized lipids. **a**, **b**, Left, scrambling is followed by the bleaching of fluorescent lipids located in the outer membrane leaflet upon the addition of the membrane-impermeable reducing agent dithionite (*). Shown are representative traces from a reconstitution containing TTYH2 (cyan) in comparison to mock liposomes of the same lipid batch (orange). Right, size exclusion chromatography profile of solubilized proteoliposomes containing TTYH2 in comparison to a sample of the protein used for reconstitution. The peak of dimeric TTYH2 is indicated by an asterisk.

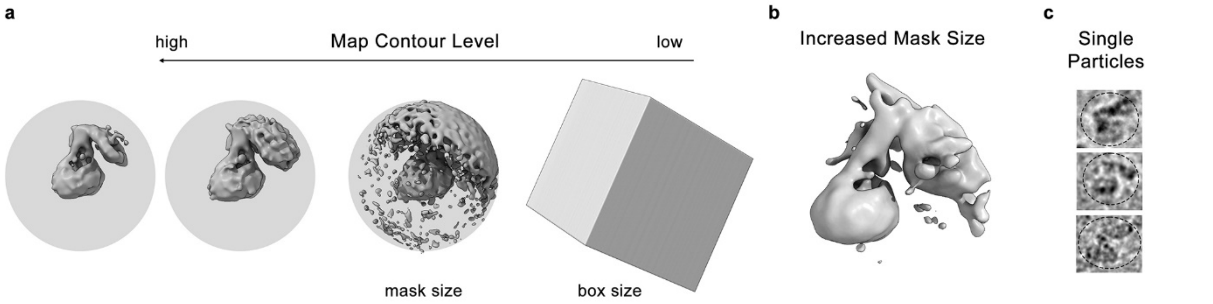

Supplementary Fig. 9. | Cryo-EM Processing of TTYH2/ApoE^{Lipidated} Data. Parameter dependence of the TTYH2-ApoE reconstruction quality. **a**, 3D reconstruction of a TTYH2/ApoE lipoprotein complex at different contour levels. At decreasing contour levels, the boundaries of the spherical mask applied during refinement become visible, and are outside of the TTYH2-ApoE complex structure. The size of the box is shown right. **b**, Density of the same reconstruction refined with a larger mask shows similar features but lower quality. **c**, Selected particle images of TTYH2 in complex with lipidated ApoE from motion and CTF-corrected micrographs.
