# Supplementary material for: Interactions between TTYH2 and APOE facilitate endosomal lipid transfer
Source: Nature. 2025 Jun 25;644(8075):273–9. doi: 10.1038/s41586-025-09200-x (PMC12328215; doi:10.1038/s41586-025-09200-x)
Supplement: Supplementary file 2 — Reporting Summary [file 41586_2025_9200_MOESM2_ESM.pdf]

nature portfolio | reporting summary

nature portfolio

Corresponding author(s): Raimund Dutzler

Last updated by author(s): May 9, 2025

Reporting Summary

Nature Portfolio wishes to improve the reproducibility of the work that we publish. This form provides structure for consistency and transparency in reporting. For further information on Nature Portfolio policies, see our Editorial Policies and the Editorial Policy Checklist.

Please do not complete any field with "not applicable" or n/a. Refer to the help text for what text to use if an item is not relevant to your study.
For final submission: please carefully check your responses for accuracy; you will not be able to make changes later.

Statistics

For all statistical analyses, confirm that the following items are present in the figure legend, table legend, main text, or Methods section.

n/a	Confirmed	
☐	☒	The exact sample size (*n*) for each experimental group/condition, given as a discrete number and unit of measurement
☐	☒	A statement on whether measurements were taken from distinct samples or whether the same sample was measured repeatedly
☐	☒	The statistical test(s) used AND whether they are one- or two-sided<br>*Only common tests should be described solely by name; describe more complex techniques in the Methods section.*
☐	☒	A description of all covariates tested
☐	☒	A description of any assumptions or corrections, such as tests of normality and adjustment for multiple comparisons
☐	☒	A full description of the statistical parameters including central tendency (e.g. means) or other basic estimates (e.g. regression coefficient) AND variation (e.g. standard deviation) or associated estimates of uncertainty (e.g. confidence intervals)
☐	☒	For null hypothesis testing, the test statistic (e.g. *F*, *t*, *r*) with confidence intervals, effect sizes, degrees of freedom and *P* value noted<br>*Give P values as exact values whenever suitable.*
☒	☐	For Bayesian analysis, information on the choice of priors and Markov chain Monte Carlo settings
☒	☐	For hierarchical and complex designs, identification of the appropriate level for tests and full reporting of outcomes
☒	☐	Estimates of effect sizes (e.g. Cohen's *d*, Pearson's *r*), indicating how they were calculated

Our web collection on statistics for biologists contains articles on many of the points above.

Software and code

Policy information about availability of computer code

Data collection	Cryo-EM images were collected using EPU 2.9 with AFIS for faster acuisition, confocal microscopy images were acquired using ZEN 2. AFM force curves were acquired using the JPK SPM 6.4.22 software.
Data analysis	Relion 4.0.4, Cryosparc 4.3 - 4.5, Chimera 1.17, ChimeraX 1.3, Phenix 1.21, Coot 0.9.8, GraphPad Prism 10, Fiji 2.14.0/1.54f, JPK-Data 6.4, JPK Data Processing 6.4, Python 3.12.

For manuscripts utilizing custom algorithms or software that are central to the research but not yet described in published literature, software must be made available to editors and reviewers. We strongly encourage code deposition in a community repository (e.g. GitHub). See the Nature Portfolio guidelines for submitting code & software for further information.

Data

Policy information about availability of data

All manuscripts must include a data availability statement. This statement should provide the following information, where applicable:

- Accession codes, unique identifiers, or web links for publicly available datasets
- A description of any restrictions on data availability
- For clinical datasets or third party data, please ensure that the statement adheres to our policy

The three-dimensional cryo-EM density maps were deposited in the Electron Microscopy Data Bank under the following accession numbers: EMD-51106 for TTYH2 in complex with Sb1, EMD-53290 for TTYH2 in complex with Sb2, EMD-53291 for TTYH2 in complex with Sb1 in cell-derived vesicles, EMD-53289 for TTYH2 in

complex with delipidated ApoE, EMD-53301 and EMD-53297 for TTYH2 complexes with coexpressed ApoE from dataset 1, EMD-53303 and EMD-53302 for TTYH2 complexes with coexpressed ApoE from dataset 2, EMD-53304 for TTYH2 complex with coexpressed ApoE from dataset 3, EMD-51108 for TTYH2 in complex with lipids, EMD-53293 for TTYH2 in complex with C-terminal ApoE fragment, EMD-53251 and EMD-53269 for TTYH2 complexes with lipidated ApoE from dataset 1, EMD-53280 for TTYH2 in complex with lipidated ApoE from dataset 2, EMD-53272, EMD-53273 and EMD-53271 for ApoE lipoprotein discs from dataset 1, and EMD-53292 for TTYH2 in complex with lipidated ApoE in cell-derived vesicles. Relevant coordinates were deposited in the Protein Data Bank under the following accession numbers: 9G6X for TTYH2 in complex with Sb1, 9G71 for TTYH2 in complex with lipids, and 9QNR for TTYH3. The cryo-EM structure of TTYH2 (PDB: 7P54) was used for building initial models of TTYH2 in complex with Sb1 and in complex with lipids and was fitted into all cryo-EM maps of TTYH2 in complex with ApoE. The structures of the GFP-binding nanobody and of TTYH3 (PDB: 7P5C) were used as initial models for building of the Sb1 and TTYH3 models, respectively. The structure of N-terminal domain of ApoE (PDB: 1B68) and the structure of TTYH2 (PDB: 7P54) were used to create illustrations in Biorender. A source data file is provided with this paper.

Research involving human participants, their data, or biological material

Policy information about studies with human participants or human data. See also policy information about sex, gender (identity/presentation), and sexual orientation and race, ethnicity and racism.

Reporting on sex and gender	N/A
Reporting on race, ethnicity, or other socially relevant groupings	N/A
Population characteristics	N/A
Recruitment	N/A
Ethics oversight	N/A

Note that full information on the approval of the study protocol must also be provided in the manuscript.

Field-specific reporting

Please select the one below that is the best fit for your research. If you are not sure, read the appropriate sections before making your selection.

☒ Life sciences ☐ Behavioural & social sciences ☐ Ecological, evolutionary & environmental sciences

For a reference copy of the document with all sections, see nature.com/documents/nr-reporting-summary-flat.pdf

Life sciences study design

All studies must disclose on these points even when the disclosure is negative.

Sample size	Sample sizes were based on standards commonly used in the field. Experiments with liposomes and binding assays were performed in multiple biological and technical replicates with similar results and further inclusion of data did not change the results. Confocal microscopy samples were prepared multiple times and similar images were obtained. For Cryo-EM datasets sufficient number of micrographs were collected to obtain best possible 3D reconstructions for each preparation type. Complete Cryo-EM statistics are provided in Extended Data Table 1.
Data exclusions	Data selection for Cryo-EM is illustrated in Extended Data Table 1 and Extended Data figures. Representative confocal microscopy images were chosen to be displayed in the manuscript. Scrambling assay data from unsuccessful reconstitutions with aggregated protein were excluded. Otherwise, no data were excluded.
Replication	Cryo-EM datasets of TTYH2 coexpressed with ApoE were acquired from three different samples resulting in similar cryo-EM maps. Cryo-EM datasets of TTYH2 in complex with lipidated ApoE were acquired from two different samples resulting in similar cryo-EM maps. Subcellular fractionation of HEK293 and N2A cells was repeated three times each with similar results. Lipid transfer assay was performed in two biological and multiple technical (TTYH2) or three technical (TTYH3) replicates and all replications were successful. Lipid transfer assay with ApoE tethering was performed in three technical replicates. Sybody displacement assay was performed in four biological and multiple technical replicates and all replications were successful. Liposomal scrambling assay was performed in two biological and four technical replicates for the destabilized preparations and in three biological and seven technical replicates for the solubilized preparation. Two of three preparations using solubilized lipids were unsuccessful as the protein aggregated during the process, the two destabilized preparations were successful. Cellular scrambling assay was performed in three biological replicates. All replications were successful for the cell-based scrambling assay.
Randomization	Randomization was used during cryo-EM data processing for the quality assessment of obtained reconstructions by randomly splitting the dataset in half, each used for obtaining a separate 3D reconstruction and measuring the correlation between the two.
Blinding	Not applicable, as we did not perform experiments requiring subjective interpretation.

Reporting for specific materials, systems and methods

We require information from authors about some types of materials, experimental systems and methods used in many studies. Here, indicate whether each material, system or method listed is relevant to your study. If you are not sure if a list item applies to your research, read the appropriate section before selecting a response.

Materials & experimental systems

n/a	Involved in the study	
☐	☒	Antibodies
☐	☒	Eukaryotic cell lines
☒	☐	Palaeontology and archaeology
☒	☐	Animals and other organisms
☒	☐	Clinical data
☒	☐	Dual use research of concern
☒	☐	Plants

Methods

n/a	Involved in the study	
☒	☐	ChIP-seq
☒	☐	Flow cytometry
☒	☐	MRI-based neuroimaging

Antibodies

Antibodies used	Antibodies purchased from ThermoFisher Scientific: rabbit anti-TTYH2 (PA5-34395), rabbit anti-ApoE (16H22L18), rabbit anti-EEA1 (F.43.1), rabbit anti-LAMP1 (107), rabbit anti-Na,K-ATPase (ST0533), rabbit anti-SERCA (JM10-20), rabbit anti-Rab7 (PA5-23138), rabbit anti-GM130 (ARC0589), mouse anti-Rab9 (Mab9), goat anti-rabbit HRP-conjugated (31460), goat anti-rabbit Alexa 488-conjugated (A-11008), goat anti-mouse Alexa 594-conjugated (A-11032). Anti-Rab11 (20229-1-AP) was obtained from Proteintech. Anti-TTYH2 antibody used for immunocytochemistry was purchased from antibodies online: https://www.antibodies-online.com/antibody/7124004/anti-Tweety+Homolog+2+TTYH2+AA+455-534+antibody/.
Validation	All primary antibodies were used for western blotting or immunocytochemistry of samples from human-derived or mouse-derived cell lines. All primary antibodies were validated by the supplier for recognition of their human-specific antigens. Rabbit anti-ApoE (16H22L18), anti-EEA1 (F.43.1), rabbit anti-Rab11 (20229-1-AP; Proteintech), rabbit anti-Na,K-ATPase (ST0533), rabbit anti-SERCA (JM10-20), rabbit anti-Rab7 (PA5-23138) and rabbit anti-GM130 (ARC0589) antibodies were also validated by the supplier for recognition of their mouse-specific antigens. Primary antibodies used for western blotting including rabbit anti-TTYH2 (PA5-34395), rabbit anti-ApoE (16H22L18), rabbit anti-EEA1 (F.43.1), rabbit anti-LAMP1 (107), rabbit anti-Rab11 (20229-1-AP; Proteintech), rabbit anti-Na,K-ATPase (ST0533), rabbit anti-SERCA (JM10-20), rabbit anti-Rab7 (PA5-23138), rabbit anti-GM130 (ARC0589) were all validated for usage in western blot analysis by the supplier and in multiple publications. The mouse anti-Rab9 (Mab9) antibody used in immunocytochemistry was validated by the supplier and in multiple publications for this application. The anti-TTYH2 antibody obtained from antibodies online that was used in immunocytochemistry was validated by western blotting by the supplier. In addition, both anti-TTYH2 antibodies and the anti-ApoE antibody were validated by us for recognition of purified recombinant proteins using western blotting.

Eukaryotic cell lines

Policy information about cell lines and Sex and Gender in Research

Cell line source(s)	HEK293T (ATCC, CLR-1573), HEK293S GnTI– (ATCC, CLR-3022), Neuro-2A (ATCC, CCL-131), HEK293 TMEM16F knockout provided by Huanghe Yang.
Authentication	No further authentication was performed.
Mycoplasma contamination	The cell lines were tested and are free from mycoplasma contamination
Commonly misidentified lines (See ICLAC register)	No commonly misidentified cell lines were used in this study.

Plants

Seed stocks	N/A
Novel plant genotypes	N/A
Authentication	N/A

This checklist template is licensed under a Creative Commons Attribution 4.0 International License, which permits use, sharing, adaptation, distribution and reproduction in any medium or format, as long as you give appropriate credit to the original author(s) and the source, provide a link to the Creative Commons license, and indicate if changes were made. The images or other third party material in this article are included in the article's Creative Commons license, unless indicated otherwise in a credit line to the material. If material is not included in the article's Creative Commons license and your intended use is not permitted by statutory regulation or exceeds the permitted use, you will need to obtain permission directly from the copyright holder. To view a copy of this license, visit http://creativecommons.org/licenses/by/4.0/
